# Transcriptome profiling of lncRNA and co-expression network in the vaginal epithelial tissue of women with lubrication disorders

**DOI:** 10.7717/peerj.12485

**Published:** 2021-11-10

**Authors:** Jingjing Zhang, Jing Zhang, Shengnan Cong, Jingyi Feng, Lianjun Pan, Yuan Zhu, Aixia Zhang, Jiehua Ma

**Affiliations:** 1 Women’s Hospital of Nanjing Medical University (Nanjing Maternity and Child Health Care Hospital), Nanjing, China; 2 Jiangsu Health Vocational College, Nanjing, China; 3 School of Nursing, Nanjing Medical University, Nanjing, China; 4 High School Affiliated to Nanjing Normal University International Department, Nanjing, China

**Keywords:** Female sexual dysfunction, Lubrication disorders, Long non-coding RNA, Co-expression network

## Abstract

**Background:**

Vaginal lubrication is a crucial physiological response that occurs at the beginning of sexual arousal. However, research on lubrication disorders (LD) is still in its infancy, and the role of long non-coding RNAs (lncRNAs) in LD remains unclear. This study aimed to explore the function of lncRNAs in the pathogenesis of vaginal LD.

**Methods:**

The expression profiles of LD and normal control (NC) lncRNAs were examined using next-generation sequencing (NGS), and eight selected differentially expressed lncRNAs were verified by quantitative real-time PCR. We conducted GO annotation and KEGG pathway enrichment analyses to determine the principal functions of significantly deregulated genes. LncRNA-mRNA co-expression and protein-protein interaction (PPI) networks were constructed and the lncRNA transcription factors (TFs) were predicted.

**Results:**

From the results, we identified 181,631 lncRNAs and 145,224 mRNAs in vaginal epithelial tissue. Subsequently, our preliminary judgment revealed a total of 499 up-regulated and 337 down-regulated lncRNAs in LD. The top three enriched GO items of the dysregulated lncRNAs included the following significant terms: “contractile fiber part,” “actin filament-based process,” and “contractile fiber”. The most enriched pathways were “cell-extracellular matrix interactions,” “muscle contraction,” “cell-cell communication,” and “cGMP-PKG signaling pathway”. Our results also showed that the lncRNA-mRNA co-expression network was a powerful platform for predicting lncRNA functions. We determined the three hub genes, ADCY5, CXCL12, and NMU, using PPI network construction and analysis. A total of 231 TFs were predicted with RHOXF1, SNAI2, ZNF354C and TBX15 were suspected to be involved in the mechanism of LD.

**Conclusion:**

In this study, we constructed the lncRNA-mRNA co-expression network, predicted the lncRNA TFs, and comprehensively analyzed lncRNA expression profiles in LD, providing a basis for future studies on LD clinical biomarkers and therapeutic targets. Further research is also needed to fully determine lncRNA’s role in LD development.

## Introduction

Female sexual dysfunction (FSD) is a complex pelvic floor disease with an incidence of 25%–63% globally and 37.6% in China (Ma et al., 2014). FSD can significantly affect women’s physical and mental health, and it can even affect their sexual partners, leading to feelings of mutual alienation (Amidu et al., 2010; Kammerer-Doak & Rogers, 2008; Laumann et al., 2005). Once a neglected subject, FSD is now being studied by a growing number of researchers who have found that its incidence is even higher than that of male sexual dysfunction (Nazareth, Boynton & King, 2003).

Vaginal lubrication is an essential physiological response during sexual arousal, and its dysfunction can lead to several problems including orgasm disorder and sexual pain (Munarriz et al., 2002). Vaginal lubrication disorder (LD), a type of sexual dysfunction that is mainly defined as the inability to elicit vulvar swelling or vaginal lubrication responses to any type of sexual stimulation, falls under the category of a genital sexual arousal disorder (Yildiz, 2015). In China, it is the most common type of FSD, accounting for approximately 97.9% of the total incidence of FSD (Ma et al., 2014). From limited literature on this subject, there is an evidence that capillary fluid in the submucosa and mucous that is secreted by the cervical and periurethral glands are involved in the vaginal lubrication process. The epithelial tissue is responsible for the transportation and rationing of ions and water molecules and plays an important role as the final gatekeeper of vaginal lubrication (D’Amati et al., 2003; Pastor & Chmel, 2018; Shabsigh et al., 1999). The role of fluid transport in vaginal lubrication, mainly in the transport of small molecules such as water, glycerol, and ions, has been explored in recent studies (Gorodeski, 2005; Sun et al., 2014). Our group identified miR-137 and its downstream effector AQP2 as important molecules involved in the regulation of vaginal lubrication (Zhang et al., 2018). Additionally, we also previously explored the differentially expressed circRNAs in women with vaginal LD, constructed a circRNA-miRNA-mRNA network, and found that hsa-miR-212-5p and hsa-miR-874-3p were associated with LD development (Cong et al., 2021; Zhang et al., 2019). These results provided some molecular basis and clues for understanding the development of vaginal LD. However, vaginal LD needs to be explored in more depth as existing treatments still leave much to be desired (Li, 2014).

Long non-coding RNA (lncRNA) is a class of endogenous non-coding RNA located in the nucleus or cytoplasm. They are the transcripts that more than 200 nucleotides long and similar to mRNA in structure but lack the ability to encode proteins (Mercer, Dinger & Mattick, 2009). Studies have shown that lncRNAs are involved in regulating epigenetic inheritance (Tsai et al., 2010), the cell cycle (Wang et al., 2019), and cell differentiation (Fatica & Bozzoni, 2014). There malfunction are closely related to the occurrence and development of tumors and diseases such as Alzheimer’s (Ponting, Oliver & Reik, 2009; Qiu et al., 2013; Yang et al., 2014). With recent developments in high-throughput sequencing and gene chip technology, some lncRNAs have been found to be closely associated with the development of vascular-derived diseases, including diabetes (Ma et al., 2020), hypertension (Shen et al., 2020), atherosclerosis, (Guo et al., 2019), coronary heart disease (Xu et al., 2020a), erectile dysfunction (Cong et al., 2020), and pre-eclampsia (Lei et al., 2021). The physiological mechanism that triggers vaginal lubrication reveal that it is dependent on vascular function, but the lncRNA profiles in vaginal LD remain unknown. Expanding our understanding of the lncRNAs involved in vaginal LD will have a profound impact on the development of FSD therapies.

The purpose of this study is to screen and study the differential expression of lncRNAs in women who have and do not have vaginal LD in order to further explore its pathophysiological mechanism and to find effective biomarkers and therapeutic targets.

## Methods & Materials

### Patient information and sample collection

Our study received approval from the Medical Ethics Committee of the Women’s Hospital of Nanjing Medical University (Ethic of Maternity [2014] No.66). The patients who participated in this study read the research purposes and methodology, then provided written consent. Taking feasibility and convenience into account, vaginal epithelial tissues were obtained from patients who were undergoing vaginal tightening procedures. Before operation, we scored and diagnosed vaginal LD based on our previous research (Ma et al., 2014). Vaginal epithelial tissues were obtained during the operation and were immediately preserved in liquid nitrogen and then transferred to 80 °C until RNA extraction. We selected six women with vaginal LD and six women without vaginal LD by convenience sampling. The subjects were all Han Chinese adult female who were planning to undergo vaginal tightening surgery, did not have other gynecological diseases, and had received secondary education or higher that can clearly understand and cooperate with the research. By the way, in view of the fact that premenopausal or menopausal women may have endocrine disorders that may lead to reduced vaginal secretions and vaginal lubrication disorders (Al-Azzawi & Palacios, 2009; Farr et al., 2015; Hall & Phillips, 2005), the patients selected for this study were young women aged around 35 years. The specimens for the transcriptomic analysis were selected from patients of similar age.

### RNA isolation

The total RNA of the vaginal epithelial tissues was extracted with Trizol (Tiangen, Beijing, China) according to the manufacturer’s instruction. RNA quality and quantification were measured using the Agilent 2100 Bioanalyzer (Agilent Technologies, Santa Clara, CA, USA). The Qubit RNA Assay Kit and the Qubit Fluorometer (Invitrogen, Waltham, MA, USA) were used for measuring the RNA concentration. The samples were set aside for follow-up experiments only when the RNA integrity number (RIN) was greater than or equal to 7.0 and when there was a 28S:18S ratio greater than or equal to 1.5. The required starting amount was between 3 to 4 ug. The starting total RNAs were accurately quantified with the Qubit RNA Assay Kit.

### cDNA library preparation and sequencing

CapitalBio Technology (Beijing, China) sequenced and generated the sequencing library, and we dislodged rRNAs from the total RNAs using the Ribo-Zero™ Magnetic Kit (Epicentre, Madison, WI, USA). Using the 5 × NEBNext First-Strand Synthesis Reaction Buffer (New England Biolabs, Ipswich, MA, USA), we determined that the RNA fragment length was ∼200 base pairs and the first strand was synthesized using reverse transcriptase and random hexam-eric primers. We then used the 10 × Second Strand Synthesis Reaction Buffer with dUTP Mix (New England Biolabs, Ipswich, MA, USA) to synthesize the second strand cDNA. Repairing the end of the cDNA fragment involved adding a single “A” base and then ligaturing the adapters. The library DNA was amplified through a polymerase chain reaction (PCR) to purify and enrich products. The final libraries were qualified and quantified using Agilent 2100 and the KAPA Library Quantification kit (KAPA Biosystems, Cape Town, South Africa). Finally, the library was paired-end sequenced on the Illumina HiSeq sequencer (Illumina, San Diego, CA, USA) with a 150-base pair reading length.

### Identifying differentially expressed genes (DEGs)

We calculated and analyzed the differential genes between the LD and control groups using the Limma package. We then used t-tests and fold-change (FC) to analyze the significance of gene expression between vaginal epithelial tissues of women with LD and the healthy control, and used the ggplot2 to package plot differential volcanoes to visualize the expression of differential genes. We used the following criteria: —log_2_FC— ≥1 and *p*-value < 0.05. The DEGs were screened using the hierarchical clustering method, and the genes with identical or similar expression behavior were clustered together.

### Quantitative real-time reverse transcription PCR

To check the sequencing data, we used quantitative real-time reverse transcription PCR (RT-qPCR) to select eight differentially expressed lncRNAs across the two groups as verification of objects. Primers were designed using Primer Premier6 (http://www.premierbiosoft.com/primerdesign/index.html) and were carried out using the Basic Local Alignment Search Tool (BLAST) from NCBI to make a unique amplification product. We also validated the expression trends of the top 20 significantly differentially expressed lncRNAs and mRNAs using RT-qPCR. Quantitative real-time RT-PCR was performed using the SYBR green operational method and the manufacturer’s comprehensive instructions. The PCR reaction system (20 µl) consisted of: 10 µl ChamQ Universal SYBR qPCR Master Mix (2 ×, Cellagen Technology, San Diego, CA, USA), 0.4 µl upstream primers (10 µmol/L), 0.4 µl downstream primers (10 µmol/L), 1.0 µl cDNA template, and ddH_2_O, repeated three times for each group. The RT-qPCR reaction conditions were: denaturation at 95 °C for 30 s, followed by 40 PCR cycles at 95 °C for 10 s, and 60 °C for 30 s. The relative gene expression levels were quantified in accordance with the cycle threshold (Ct) values and were suitably normalized to the GAPDH internal parameter using the 2^−ΔΔ*Ct*^ (Livak) method (Schmittgen & Livak, 2008).

### Gene ontology (GO) and pathway analysis

We used GO analysis to determine the functional properties of DEGs. The candidate genes were used to cast light upon GO terms in the database (http://www.geneontology.org/). Pathway analysis can be used to show the DEGs in the Kyoto Encyclopedia of Genes and Genomes (KEGG) pathways. The *p*-value showed the significance of the pathways and GO terms; the threshold value of *P* is 0.05 and a lower *p*-value represents a more significant pathway or GO term.

### Construction of lncRNA-mRNA co-expression network

The construction of a co-expression network of the coding-non-coding gene intuitively showed the correlation between lncRNAs and mRNAs. Pearson correlation analysis was used on the expression of differentially expressed lncRNAs and mRNAs, and we selected the lncRNA-mRNA pairs with absolute values of Pearson correlation coefficient (—PCC—)>0.99 and *P*<0.05. Cytoscape software was used to map the lncRNA-mRNA co-expression network.

### Construction of protein-protein interaction networks

STRING (version 11.0, http://string-db.org) is an online tool for evaluating protein-protein interaction (PPI) information. Cytoscape is commonly used to visualize complex networks and its plug-in, CytoHubba, can be used to calculate gene yield values and screen for hub genes. The 499 DEGs were first imported into STRING to obtain their interactions, and then the remaining interactions were imported into Cytoscape. We used a combined score for the protein relationships. When the combined score between the two proteins was greater than the set threshold, the pair was extracted if both proteins were significantly different in the comparison. The top 10 genes were calculated from the protein interaction network using CytoHubba and the degree algorithm, the MCC algorithm, the EPC algorithm, and the MNC algorithm. Finally, the top 10 genes from all four algorithms were selected as the core genes after taking the intersection from the Venn diagram. We used the SPSS 26.0 to plot ROC curves and evaluated the diagnostic performance of each core gene by calculating the area under the ROC curve (AUC). A core gene was considered to have good diagnostic performance when the AUC value was >0.7.

### Transcription factor (TF) prediction for differential lncRNAs

Specific binding of TFs to regions regulating gene expression was an important mode of gene expression regulation. TF prediction was performed using the JASPAR database and TFBSTools, which gave the binding site, direction, and scoring of TFs within regions 2,000 bp upstream and 500 bp downstream from the start of each lncRNA.

### Western blot

Total protein was obtained using RIPA buffer with cocktail inhibitors (Cell Signaling Tech, USA). Protein concentration was measured using a BCA kit (Pierce, USA). Equal amounts of protein were separated with PVDF membranes (AmerSham, USA). The membranes were blocked in 5% skim milk in Tris Buffered Saline with Tween^®^ 20 (TBST) for 2 h and then incubated overnight at 4 °C with primary antibodies. The membranes were washed with TBST by three times and incubated with horseradish peroxidase-conjugated secondary antibody for 2 h at room temperature. Blots were developed using a chemiluminescence kit (Pierce, USA) and exposed to X-ray film.

### Data analysis

FastQC was employed to evaluate the quality of raw data sequencing in fastqc format, and NGSQL was used to filter the low-quality data. With the default parameters of Tophat2, the high-quality clean reading was aligned to the reference genome, using the human genome HG19 (UCSC) as a reference genome. We used Cufflinks and Cuffmerge software on the assembly of transcripts. Using the transcript results, all lncRNAs and mRNAs were compared and analyzed. mRNA and lncRNA transcripts were processed as known mRNAs or lncRNAs and annotated using the public database. When forecasted to be a non-coding RNA, the sequence length was greater than 200, and the new transcript was deemed as a new lncRNA. We used limma package and edgeR to carry out differential expression analysis. Based on the sequence similarity and location on the reference genome, we applied cis- and trans- patterns to forecast the lncRNA target genes.

## Results

### General characteristics of the lncRNA and mRNA in vaginal LD

**Figure 1:**
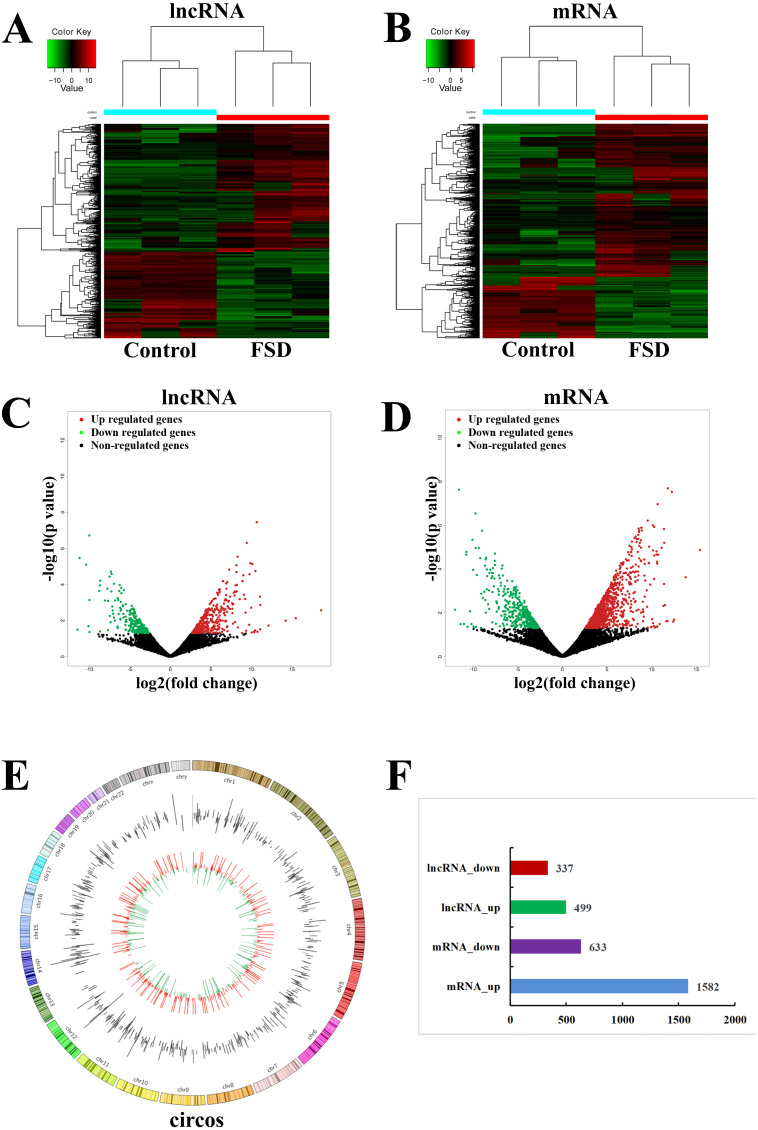
LncRNA and mRNA expression changes profiles in vaginal lubrication disorders (LD). (A & B) The hierarchical clustering analysis of significantly up-regulated or down-regulated lncRNAs and mRNAs, respectively. (C & D) The scatterplots for differentially expressed lncRNAs and mRNAs, respectively. The scatterplots were generated using——log_2_FC——≥ 1 and *p*-value < 0.05. The red dots in the figure represent the up-regulated lncRNAs and mRNAs that are statistically significant, while the green dots represent the down-regulated ones. (E) Distrubution of all identified circRNAs between the LD and NC groups. (F) The number of differentially expressed lncRNAs and mRNAs.

Based on the quality-controlled of the authoritative databases, 181,631 lncRNAs and 145,224 mRNAs in 6 lubrication disorder samples and paired healthy samples were constructed. The hierarchical clustering (Figs. 1A and 1B) and scatterplot (Figs. 1C and 1D) showed the differential expression of lncRNAs and mRNAs between LD group and the control group. In comparison with the control, we confirmed that 499 lncRNAs were up-regulated and 337 were down-regulated in all in LD (—log_2_FC— ≥1 and *p*-value < 0.05) (Fig. 1F). The top 20 significantly deregulated lncRNAs are shown in Table 1. It was shown that the expression levels of differentially expressed lncRNAs were fully capable of differentiate lubrication disorder samples and normal samples. The top 20 significantly deregulated mRNAs are shown in Table 2. Figure 1E showed the distribution of all identified lncRNAs between LD and NC groups. Furthermore, these up- and down- upregulated lncRNAs were spread over the genome and overlapped all chromosomes. These observations suggest that the landscape of the entire transcriptome during LD development may be reshaped by a potential dynamic interaction between lncRNAs and coding RNAs.

**Table 1:** The top 20 deregulated lncRNAs in lubrication disorders (LD).

**LncRNA**	**Regulation**	**Track gene**	**LogFC**	***P*** **value**	**Preidicted TFs**	**qPCR fold change**
MERGE.13308.8	up	MERGE.13308	18.59021095	2.70E−03	DLX6, RBPJ, NFATC2, BSX, UNCX, MZF1, ISX, MSX2, RAX2, RHOXF1, NFIX, PRRX1, NFIA, BARX1, SHOX, ZNF354C, STAT3	9.226425384
MERGE.14929.5	up	MERGE.14929	15.45704606	7.38E−03	MZF1, TCF3, ZNF384, FOXL1, SNAI2, SPIB, TFE3, TEAD3, TCF4, RHOXF1	7.479190782
NONHSAT234217.1	up	MERGE.17169	14.22167555	1.02E−02	ZNF354C, DLX6, MEIS1	4.986763369
MERGE.29036.1	up	MERGE.29036	12.08543036	1.90E−02	KLF16, SP1, SP8, RHOXF1, SP3, DLX6, SPIB, MEIS1, MZF1	5.037770479
MERGE.62067.1	up	MERGE.62067	11.08023213	1.37E−03	MEIS1, RAX2, MSX1, FOXD2, RHOXF1, EN1, NFATC2, FOXI1, FOXG1, FOXP3, KLF5, BARX1, FOXL1, ISX, ZNF384, LBX2, ZNF354C, MSX2, FOXD1, FOXO3, SHOX, FOXO4, BSX, MYB, SPIB, MZF1, FOXO6, DLX6, LHX9	5.631547226
NONHSAT258741.1	up	MERGE.62035	11.06269073	3.08E−02	FOS, TCF3, BARX1, SNAI2, MEIS1, MZF1, ZNF740, VAX2, SPIB, ZNF354C, VAX1, RUNX3, FIGLA, RHOXF1, MNX1, NKX6-2, HOXA5, PDX1, CDX1, MYB, DLX6, NKX6-1	4.686123849
MERGE.19926.6	up	MERGE.19926	11.04714822	4.75E−04	ZNF384, TCF3, NFYA, TCF4, KLF5, HIF1A, THAP1, BARX1, MEIS1, OTX2, SP1, ZNF354C, RHOXF1, MZF1, ARNT::HIF1A	2.970739229
MERGE.25323.3	up	MERGE.25323	10.73936911	4.19E−02	NR4A2, SNAI2, FOSL1, MZF1, RBPJ, NFIA, NFIX, JUNB, ZNF354C, MYB, HIC2, FOSL2, RHOXF1	4.04161483
MERGE.59325.11	up	MERGE.59325	10.64785935	3.51E−08	MSX2, GATA5, UNCX, KLF5, RAX2, TBX15, MGA, PRRX1, TBX1, CEBPA, LHX9, TBX5, ZNF384, FIGLA, MEIS1, ISX, MZF1, RHOXF1, BARX1, TBX4, MSX1, NFATC2, ZNF354C, DLX6, POU3F4, SHOX, BSX, SPIB	3.606962335
NONHSAT227526.1	up	MERGE.5169	10.47424434	1.79E−05	NKX6-2, VAX1, TEAD3, BSX, TBX15, VAX2, MZF1, TBX1, SREBF1, FOS, RBPJ, TBX4, FOXG1, NFIX, KLF5, TBX5, SREBF2, NKX6-1, MSX1, MSX2, SP1, FOXL1, TBX21, ZNF354C, DLX6, NFIA, PDX1, RHOXF1, MEIS1, MGA	2.113818893
MERGE.19364.3	down	MERGE.19364	−11.4609105	3.18E−02	HOXB3, VAX2, VSX1, NFIA, TFAP2C (var.2), ZNF384, PDX1, RHOXF1, DLX6, YY1, GATA5, GATA3, STAT3, TFAP2A, ZNF354C, VSX2, TFAP2B (var.2), VAX1, EN1, NR4A1	0.250934797
NONHSAT094312.2	down	MERGE.42993	−11.2139755	3.36E−06	BHLHE22, MYB, RELA, RHOXF1, MZF1, GATA5, BHLHE40, FOXL1, ZNF354C, NFIA, NFIX, TFE3, GATA3, SPIB, GATA6, MEIS1	0.216712247
NONHSAT237433.1	down	MERGE.26394	−10.4010082	7.94E−06	NKX2-8, ZNF354C, MZF1, RHOXF1, FOXH1, ZNF384, SPIB, NFATC2, FOXO6, NFIX, FOXL1, FOXP3, GATA5, FOXI1, FOXO4, BARX1, GATA3, MEIS1, FOXD2, OTX1, OTX2, PITX3, HOXA5	0.37435834
MERGE.22093.11	down	MERGE.22093	−10.112809	2.06E−02	DLX6, MEIS1, LHX9, RAX2, MSX1, UNCX, MIXL1, BARX1, LBX1, MSX2, ISX, BSX, PDX1, OTX2, ZNF354C, ZNF384, SHOX, NKX6-2, NKX6-1, RHOXF1, MYB, TCF4, TCF3, MNX1, PRRX1, HOXA5, MZF1	0.263678391
MERGE.13442.1	down	MERGE.13442	−10.0134944	1.90E−07	NFATC2, SPIB, ZNF384, MEIS1, MZF1, KLF5, RHOXF1, MEIS3, STAT3, MEIS2, ZNF354C, STAT1	0.520026317
MERGE.50943.3	down	MERGE.50943	−9.99578708	4.32E−02	BARX1, FOXO4, MEIS3, RHOXF1, ZNF354C, UNCX, FOXI1, ZNF384, MEIS2, TEF, MEIS1, LMX1A, FOXG1, FOXO3, FOXD1, FOXP3, FOXD2, ISX, LMX1B, FOXO6, SHOX, NFATC2, MIXL1, MZF1, FOXL1	0.425092965
MERGE.43176.6	down	MERGE.43176	−9.98923044	7.24E−04	ID4, NFIA, MYC, KLF5, SPIB, TEAD3, ZNF354C, SNAI2, MEIS1, SP1, NFIX, MZF1	0.247329872
MERGE.791.7	down	MERGE.791	−8.79298043	2.26E−04	GATA5, FOXO6, SPIB, FOXO4, KLF5, MYB, RHOXF1, FOXI1, TCF4, FOSL2, FOXD2, ARNT::HIF1A, NRF1, GATA3, MZF1, FOSL1, SP1, NFIX, JUNB, ZNF354C, FOXL1, FOXP3	0.431813425
NONHSAT227852.1	down	MERGE.5992	−8.69413796	1.13E−04	TEAD3, SP1, SOX13, RHOXF1, KLF5, MEIS3, GSC2, GSC, MEIS2, PITX3, GATA3, TEAD2, TFE3, NRL, MZF1, NR4A1, SOX9, HOXC10, TEAD4, MYB, FOXL1, GATA5, MEIS1	0.624916621
MERGE.35898.5	down	MERGE.35898	−8.67534141	3.99E−02	TFDP1, TEAD3, E2F4, RBPJ, ZNF384, E2F6, ZNF354C, MZF1, RHOXF1	0.315777779

**Table 2:** The top 20 deregulated mRNAs in lubrication disorders (LD).

**mRNA**	**Track gene**	**Regulation**	**Symbol**	**Pathway term**	**GO term**	**LogFC**	***P*** **value**	**qPCR fold change**
ENST00000618621	MERGE.42841	up	LPP	–	cell adhesion *etc.*	15.39594	1.35E−05	7.07218
ENST00000554617	MERGE.17909	up	FOS	cAMP signaling pathway *etc.*	reproduction *etc.*	13.79122	2.39E−04	7.82350
ENST00000524189	MERGE.56201	up	KIF13B	vesicle-mediated transport *etc.*	cell morphogenesis *etc.*	12.50214	2.04E−02	6.71365
ENST00000378933	MERGE.61388	up	TAB3	NF-kappa B signaling pathway *etc.*	MAPK cascade *etc.*	12.41181	2.53E−02	5.76634
ENST00000495522	MERGE.54887	up	CALD1	vascular smooth muscle contraction *etc.*	muscle system process *etc.*	12.25227	2.98E−08	6.16173
ENST00000604624	MERGE.7522	up	KCNMA1	Ca ^2+^ activated K ^+^ channels *etc.*	response to hypoxia *etc.*	11.80386	2.05E−08	5.53297
ENST00000535737	MERGE.62662	up	FHL1	staphylococcus aureus infection *etc.*	immune effector process *etc.*	11.76909	2.35E−02	4.97539
ENST00000316292	MERGE.50636	up	EEF1A1	RNA transport *etc.*	nucleobase-containing compound metabolic process *etc.*	11.63237	2.42E−02	3.72391
ENST00000290378	MERGE.19073	up	ACTC1	Cardiac muscle contraction *etc.*	muscle contraction *etc.*	11.48328	4.44E−03	3.69505
ENST00000530866	MERGE.10267	up	LTBP3	elastic fibre formation *etc.*	intracellular protein transport *etc.*	11.40485	6.35E−06	4.08425
ENST00000438362	MERGE.3267	down	CSDE1	–	reproduction *etc.*	−12.0215	3.18E−02	0.29101
ENST00000588188	MERGE.26410	down	PRKAR1A	insulin signaling pathway	actin cytoskeleton organization *etc.*	−11.5882	3.36E−06	0.37288
ENST00000555572	MERGE.25926	down	NME1-NME2	metabolic pathways *etc.*	epidermis development *etc.*	−11.4146	7.94E−06	0.42573
ENST00000439383	MERGE.42731	down	PSMD2	epstein-barr virus infection *etc.*	morphogenesis of an epithelium *etc.*	−11.0173	2.06E−02	0.45590
ENST00000398514	MERGE.48061	down	DPYSL3	pyrimidine metabolism *etc.*	response to stimulus *etc.*	−10.7505	1.90E−07	0.30212
ENST00000487676	MERGE.49859	down	HLA-DQB1	cell adhesion molecules (CAMs) *etc.*	cell activation *etc.*	−10.7313	4.32E−02	0.39846
ENST00000342216	MERGE.29023	down	PKN1	PI3K-Akt signaling pathway *etc.*	activation of MAPK activity *etc.*	−10.6141	7.24E−04	0.31874
ENST00000354956	MERGE.39633	down	ATG7	regulation of autophagy *etc.*	cellular response to stress *etc.*	−10.3407	2.26E−04	0.17802
ENST00000468064	MERGE.35761	down	D2HGDH	metabolism *etc.*	organic acid metabolic process *etc.*	−10.1826	1.13E−04	0.50124
ENST00000393893	MERGE.10452	down	CORO1B	–	regulation of smooth muscle cell migration *etc.*	−10.0713	3.99E−02	0.59471

### Verification of candidate dysregulated lncRNAs

**Figure 2:**
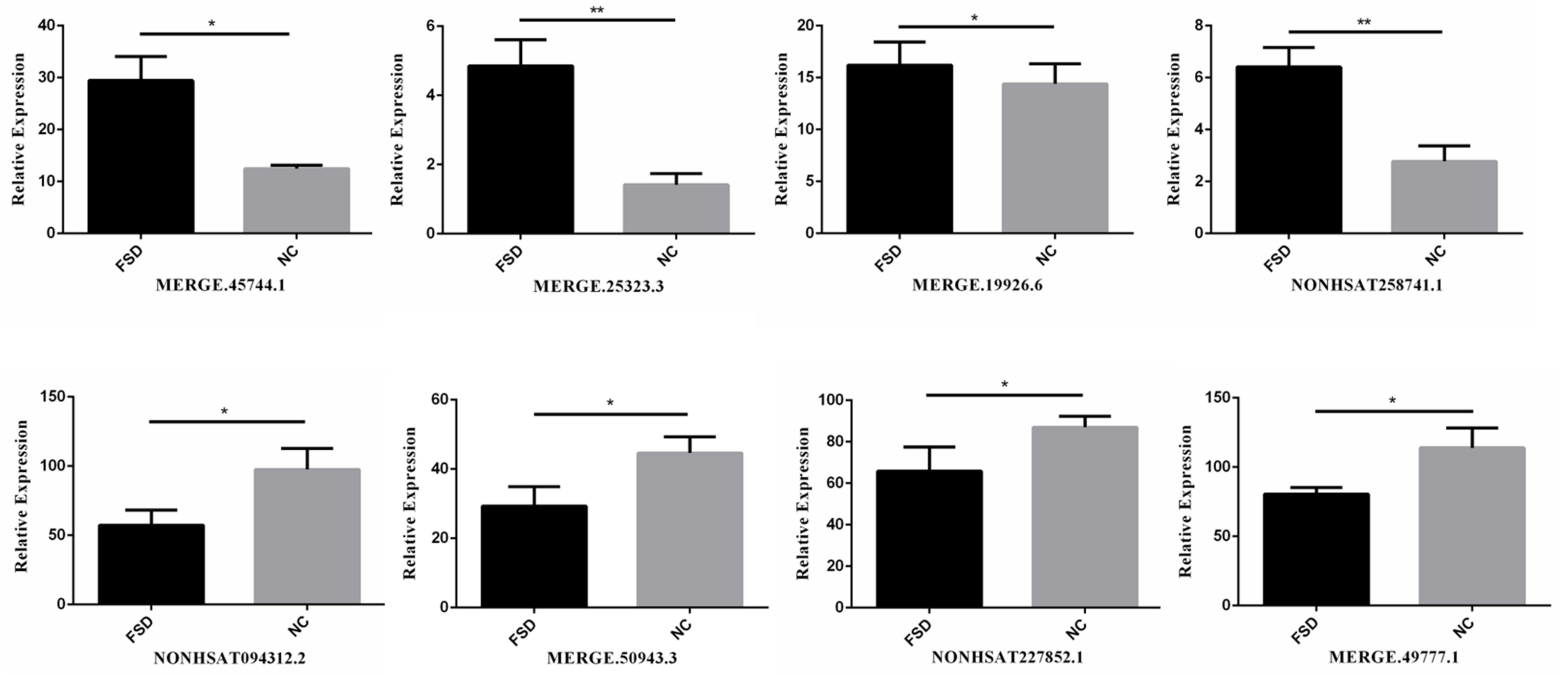
RT-qPCR for the expression verification of eight lncRNAs. Four lncRNAs were up-regulated and four lncRNAs were down-regulated in FSD, and the results were consistent with the sequencing data. *: *P* value < 0.05; **: *P* value < 0.01.

We validated the transcriptome results using an additional 6 specimens (3 LD *vs.* 3 NC). Eight differentially expressed candidate lncRNAs from the sequencing data, including four up-regulated lncRNAs (MERGE.45744.1, MERGE.25323.3, MERGE.19926.6, and NONHSAT258741.1) and four down-regulated lncRNAs (NONHSAT094312.2, MERGE.50943.3, NONHSAT227852.1, MERGE.49777.1), were selected to validate through RT-qPCR. All selected lncRNAs have an expressed fold-change of over 7 and the primers used in this study are shown in Table 3. The results of RT-qPCR showed that the expression levels of eight lncRNAs were consistent with sequencing data (Fig. 2). We also validated the top 20 differentially expressed lncRNAs and 20 mRNAs using RT-qPCR, the results are displayed in Tables 1 and 2.

**Table 3:** Primers used in this study.

Gene name	Forward (5′–3′)	Reverse (5′–3′)	Annealing temperature (° C)	Aim band length (bp)
MERGE.45744.1	ACTTTTATCTTTCCTGTCCATCA	CCCTGCTCCAACTTCCATA	60	194
MERGE.25323.3	ACGATGGCAAGGTGGTGTC	GATCTTCCAGTGGGATCTGTG	60	117
MERGE.19926.6	CACCGAGGCACATTTGAA	AGACACCACAGAGCTAAGGCT	60	121
NONHSAT258741.1	TAACCCTTCCACTCCCTTTGT	TAGGTAACCAGCACCCTCTTG	60	163
NONHSAT094312.2	ACCTTGACCTCTGTCCCTCTT	GTATGCTCTGTGGCTTGCTG	60	129
MERGE.50943.3	GCTGGCTGGTGACTGTCCT	AATCGGCTTCCATTTCTTG	60	140
NONHSAT227852.1	GCTATCTGGACCCTGCTCA	GGGCTCATTCCTTTGCTCT	60	131
MERGE.49777.1	CGGGACACGGCGGTGTAGA	GGTCGGGAGGGAAATGGC	60	159
GAPDH	GGACCTGACCTGCCGTCTAG	GTAGCCCAGGATGCCCTTGA	60	100

### GO and pathway analysis

**Figure 3:**
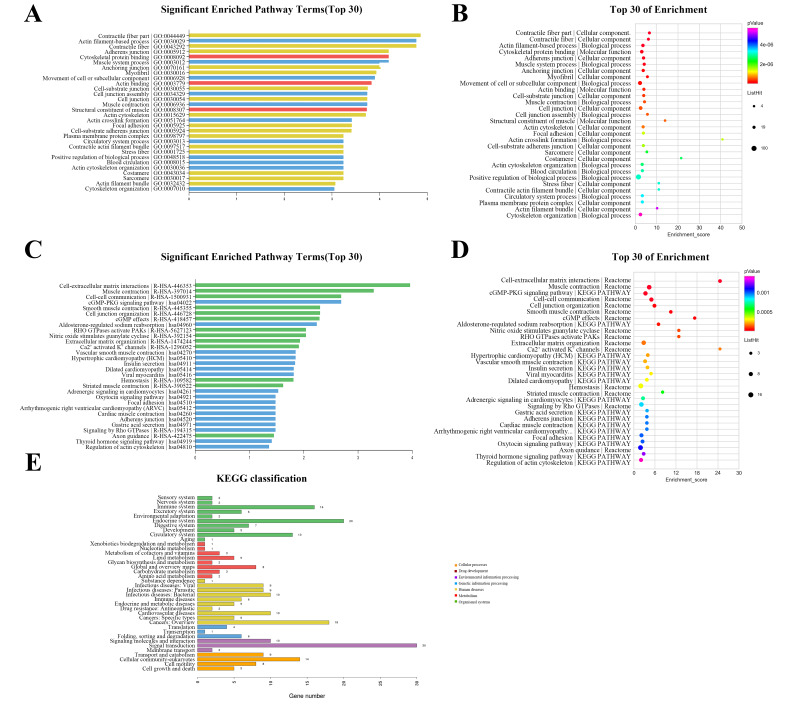
Gene ontology (GO) classification and pathway analysis of differentially expressed lncRNAs. (A) The top 30 enriched GO items of dysregulated lncRNAs. (B) Bubble chart intuitively illustrating the rich factors of the top 30 GO terms. (C) The top 30 enriched pathway items of the dysregulated lncRNAs. (D) Bubble chart intuitively illustrating the rich factors of the top 30 pathway terms. (E) A total of 263 genes were involved in 38 differential classifications in the KEGG pathway analysis.

The GO annotation describes gene and gene product attributes in humans and other organisms (http://www.geneontology.org). The three GO domains are: cellular component, biological process and molecular function. Using GO analysis on the DEGs, we found that cellular process (ontology: biological process), cell (ontology: cellular component), and binding (ontology: molecular function) were the terms with the most genes. The top 30 enriched GO items of dysregulated lncRNAs are shown in Fig. 3A, with the top three significant terms being contractile fiber part (GO:0044449), actin filament-based process (GO:0030029), and contractile fiber (GO:0043292). The bubble chart (Fig. 3B) illustrated the rich factors of the top 30 GO terms. Actin crosslink formation (GO:0051764) had the greatest enrichment. Additionally, we analyzed pathway annotation and enrichment of DEGs to further understand the biological functions of genes based on pathway analysis (Figs. 3C–3E). The first 30 enrichment pathways are organized in Figs. 3C and 3D based on their *p*-values. The most enriched pathways were “cell-extracellular matrix interactions,” “muscle contraction,” “cell–cell communication,” and “cGMP-PKG signaling pathway”. “Cell-extracellular matrix interactions” and “Ca^2+^ activated K^+^ channels” were the pathways with the maximum enrichment score. Ultimately, 263 genes were involved in 38 differential classifications in the KEGG pathway analysis (Fig. 3E). Among these classifications, signal transduction contained the most genes.

### Construction of the lncRNA-mRNA co-expression network

**Figure 4:**
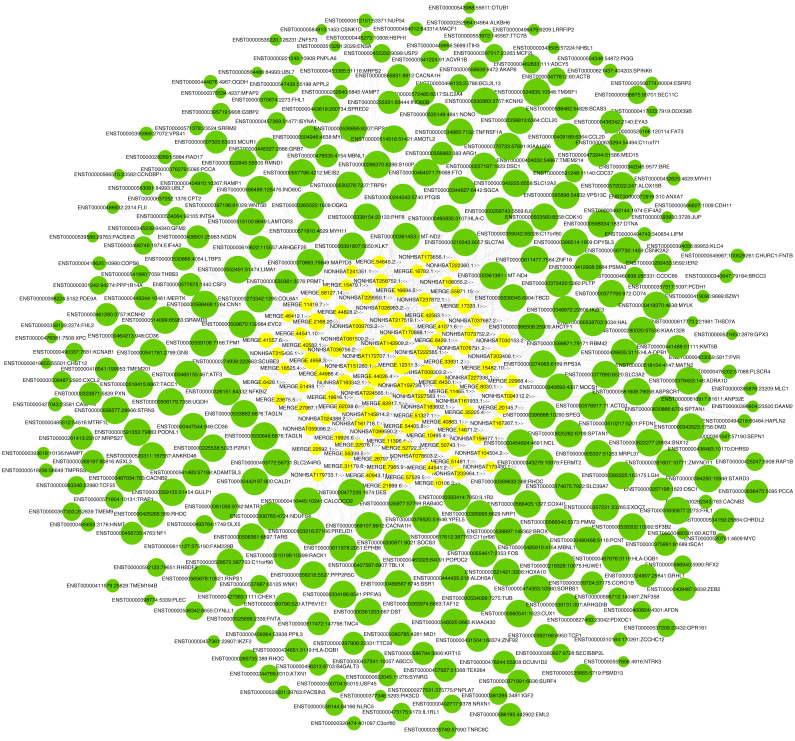
Co-expression network consisting of 100 lncRNAs and 311 mRNAs. Yellow nodes represent lncRNAs, and the larger the node, the more connections. Green nodes represent mRNAs; the smaller the node, the more connections. The edges between lncRNAs and mRNAs represent their relationships. Among the lncRNAs, NONHSAT172707.1, MERGE.44026.4, NONHSAT142909.2, and MERGE.12311.3 had the most ligands. Among the mRNAs, ESRP2, MLC1, PNPLA6, MYH11, HAPLN2, ANP32E, PACSIN3, and TMEM184B all had eight ligands.

**Figure 5:**
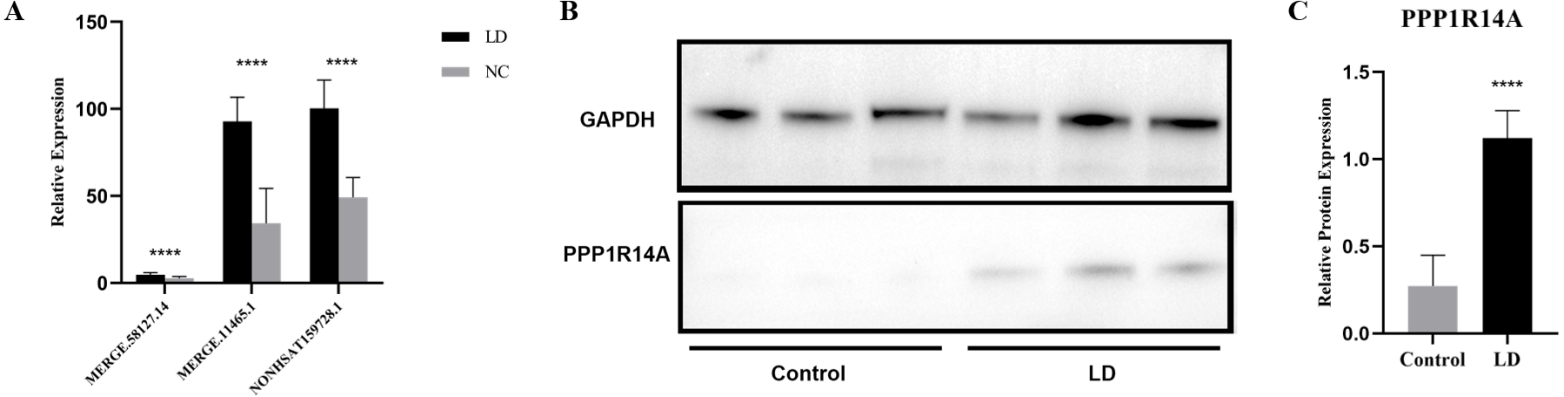
Validation for MERGE.11465.1, MERGE.58127.14, NONHSAT159728.1 and PPP1R14A. (A) RT-qPCR validation results for the differential genes MERGE. 11465.1, MERGE.58127.14, and NONHSAT159728.1. (B, C) Western Blot detects the expression of PPP1R14A protein. ****: *P* value 0.0001.

LncRNA has been reported to play an important role in a variety of gynecological diseases, including polycystic ovary syndrome (Zhao et al., 2019), endometriosis (Panir et al., 2018), and gynecological tumors. The co-expression network of lncRNA and mRNA is of great significance for the preliminary prediction of lncRNA function and can provide evidence for the participation of lncRNA in core biological functions (Mo et al., 2019). In this study, we constructed a co-expression network based on the normalized signal intensity of misaligned lncRNA and target mRNA. We also intuitively studied the relationship between the different expressions of lncRNAs and their significantly related mRNAs. The gene correlation was calculated using the Pearson algorithm, and the correlation coefficient and *p*-value were obtained. If the absolute value of the correlation coefficient was more than 0.99 and the *p*-value was less than 0.05, a co-expression relationship between lncRNAs and mRNAs was suggested. We used miRanda software to predict the lncRNA-mRNA relationships. A total of 100 lncRNAs and 311 mRNAs were included in the 411 network nodes that made up the co-expression network. They interacted to build 765 connecting bridges (Fig. 4). Notably, all relationship pairs in this co-expression network were negatively correlated. Through this co-expression network, we found that one lncRNA can target up to 17 coding genes, and a single coding gene can be associated with up to eight lncRNAs. Western blot was used to verify PPP1R14A expression and RT-qPCR was used to verify the expression of three co-expressed lncRNAs, including MERGE.11465.1, MERGE.58127.14, and NONHSAT159728.1, with the results consistent with our predictions (Fig. 5).

### PPI network analysis

**Figure 6:**
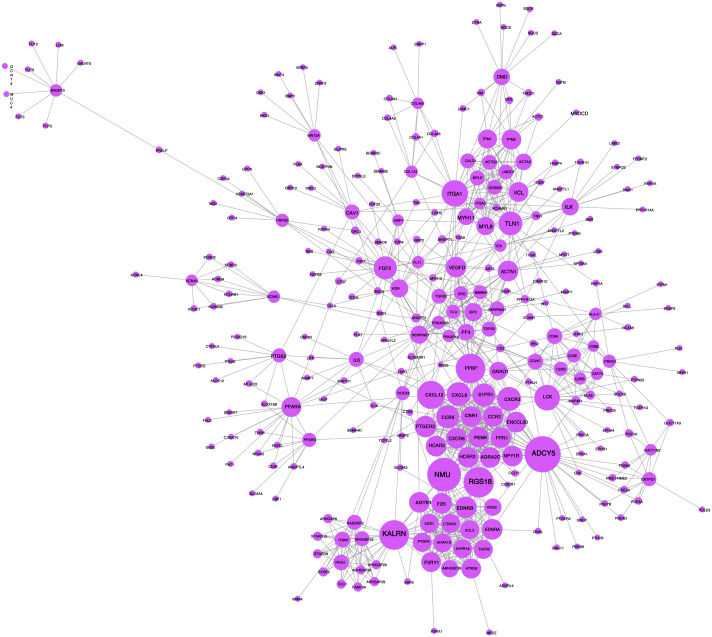
PPI network analysis graph. Node size represents clustering coefficient. Larger node size represents a larger clustering coefficient. The top ten proteins with the highest node degrees were ADCY5, NMU, RGS18, KALRN, PPBP, CXCL12, ITGA1, LCK, CXCR3, and TLN1.

**Figure 7:**
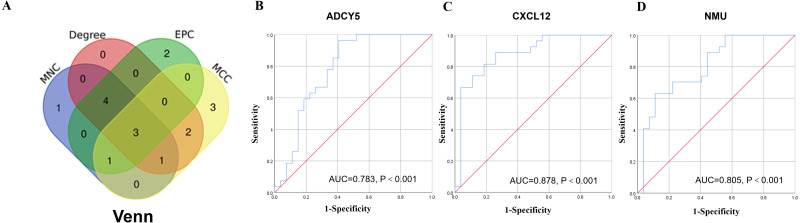
Core gene screening and ROC curve of core gene diagnosis LD. (A) The Venn diagram was drawn using the four algorithms: degree, MCC, EPC, and MNC. The three genes intersected by the four algorithms were used as hub genes . (B, C and D) ROC curves for ADCY5, NMU and CXCL12 in distinguishing vaginal lubrication disorders and normal. Abscissa represents specificity and ordinate represents sensitivity.

A network analysis of protein interactions was carried out on 499 DEGs using the STRING database, and the results were imported into Cytoscape software where the protein relationships were scored using the combined score. The resulting interaction network consisted of 289 nodes and 959 edges (Fig. 6). The cytoHuhha was used to analyze the core genes and the top 10 DEGs were considered as core genes across all three algorithms. The final core genes for vaginal LD, obtained by Venn diagram intersections, were ADCY5, CXCL12, and NMU (Fig. 7A). Based on the 27 LDs *vs* 27 NCs data results, we plotted the ROC curves for the 3 core genes using SPSS 26.0. The results are shown in Figs. 7B, 7C and 7D. The screened genes ADCY (AUC = 0.783), CXCL12 (AUC = 0.878) and NMU (AUC = 0.805) have relatively high diagnostic value.

### Prediction of TFs

The specific binding of TFs to the regulatory regions which confer gene expression is an important gene transcription regulation mechanism. Predicted TFs of abnormally expressed lncRNAs can bind to lncRNA promoters to regulate the expression of lncRNA, thus affecting the key components of disease pathogenesis (Huang et al., 2017; Jiang et al., 2018; Long et al., 2017). Based on the lncRNA-mRNA relationship pairs obtained by co-expression analysis, we used the JASPAR database (http://jaspar.genereg.net/) and TFBSTools to predict the TFs. They also allowed us to obtain the upstream of each lncRNA-TF binding site, direction and scoring in the region 500 bp downstream of  2,000  bp.

**Figure 8:**
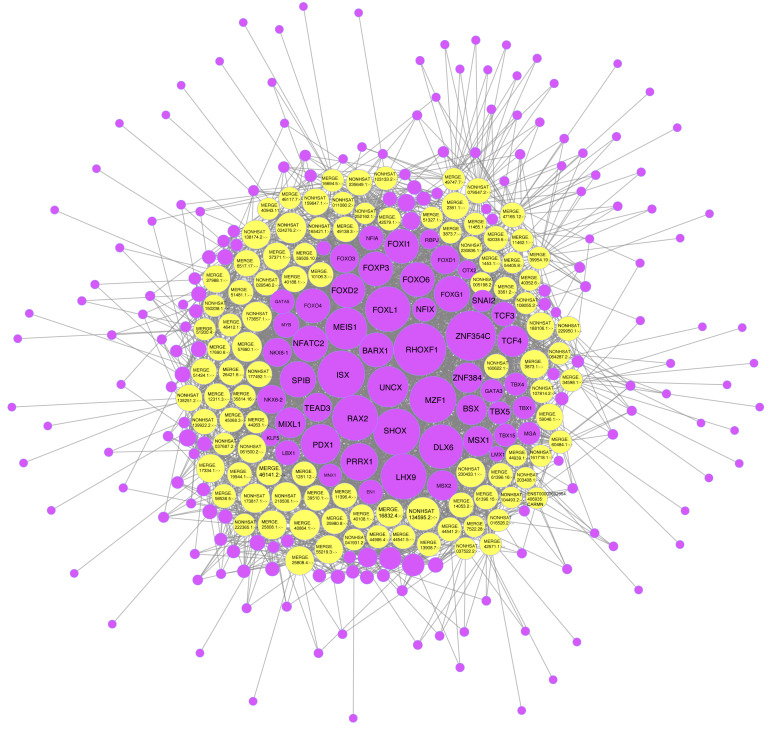
One hundred lncRNAs in the network and the predicted transcription factors (TFs). The yellow dots refer to the different lncRNAs, and the purple dots refer to the TFs. The larger the dot, the more nodes connected to it. The lncRNA with the most nodes connected to it was NONHSAT134595.2 with 53 nodes connected to it. Those connected to more than 50 nodes in the TFs included RHOXF1, ZNF354C, ISX, MZF1, and SHOX.

We found that 100 lncRNAs in the co-expression network were predicted to be paired with 231 TFs (Fig. 8). Among these, NONHSAT134595.2 matched a total of 53 TFs, including RHOXF, TBX15, and FOXD1. These TFs consisted of eight classes: “C2H2 zinc finger factors,” “Basic helix-loop-helix factors (bHLH),” “Fork head/winged helix factors,” “Homeo domain factors,” “Rel homology region (RHR) factors,” “Other C4 zinc finger-type factors,” “SMAD/NF-1 DNA-binding domain factors,” and “T-Box factors”. Of the 53 TFs related to NONHSAT134595.2, the RHOXF1 was correlated with up to 92 lncRNAs. Additionally, NONHSAT024276.2, MERGE.40864.1, MERGE.57680.1, NONHSAT173657.1, NONHSAT218506.1, NONHSAT173817.1, and NONHSAT230433.1 were associated with more than 45 lncRNAs.

## Discussion

An LD is defined as the inability to attain a sufficient lubrication response during sexual activity (Sawatsky, Dawson & Lalumière, 2018). It not only causes pain and obstacles to orgasm during sex, but it can also be the cause of interpersonal conflict (Isbill, 2018). Research on vaginal LD is still in the initial stages and no explicit targeted diagnosis or treatments have been proposed (Miller et al., 2018; Weinberger et al., 2019). Current treatments mainly rely on hormone replacement therapy (HRT), drug, or psychotherapy, but effects are not significant for all patients and vary in effectiveness and practicability (Weinberger et al., 2019).

In recent years, more researches on lncRNAs have been conducted. Many previously unknown lncRNAs have been identified at an unprecedented rate, and their significance in the diagnosis and treatment of diseases have received great attention (Lin, 2020; Xu et al., 2020b). LncRNAs are involved in transcriptional activation and inhibition (Liu et al., 2018), embryonic (Yan et al., 2013) and tissue development (Schmitz, Grote & Herrmann, 2016), and many other activities. A striking feature of lncRNAs is that they are expressed in a more tissue-specific manner than protein-coding RNAs (Deniz & Erman, 2017). Possible physiological causes of vaginal LD include endocrine disorders, vascular endothelial damage, and nervous system abnormalities (Bergh & Giraldi, 2014; Courtois et al., 2018; Diehl et al., 2016; Imprialos et al., 2018; Salonia et al., 2010; Salonia et al., 2006; Wierman et al., 2010). Studies have shown that some lncRNAs are closely related to lesions of nerves (Zhao et al., 2013), blood vessels (Simion, Haemmig & Feinberg, 2019), vaginal smooth muscles, and vaginal epithelium (Wei et al., 2020). In previous research, next-generation sequencing was conducted to mine differentially expressed circRNAs in the vaginal epithelial tissue of women with vaginal LD, and circRNA-miRNA-mRNA networks were conducted (Camilleri, Sandler & Peery, 2020; Cong et al., 2021; Zhang et al., 2019). Few studies have explored the relationships between lncRNA and vaginal LD. Therefore, we investigated the differential expression of lncRNAs in order to explore its possible mechanisms.

Using next-generation sequencing, we detected a total of 21,368 lncRNAs and 48,806 mRNAs. We identified 499 up-regulated and 337 down-regulated lncRNAs with 1,582 up-regulated and 633 down-regulated mRNAs in LD tissue compared to healthy vaginal epithelial tissue with a filter of —log_2_FC— ≥1 and a *p*-value < 0.05. GO and pathway analysis provided more information about the function of the target genes. Dysregulated lncRNAs were closely interrelated with few important biological processes connected with the LD mechanism, including muscle contraction, structural constituent of muscle, and blood circulation.

Since most of the lncRNAs functioned through the regulation of mRNAs, the lncRNA-mRNA co-expression network provided us with a powerful platform for the prediction of lncRNA function (Iwakiri, Terai & Hamada, 2017). Using the lncRNA-mRNA co-expression network, we found that three lncRNAs (MERGE.11465.1, MERGE.58127.14, and NONHSAT159728.1) were connected with PPP1R14A, which was functionally related in LD tissue. It has been found that the phosphorylation of PPP1R14A can suppress the function of myosin phosphatase, thereby affecting smooth muscle contraction (Lartey et al., 2007; Sakai et al., 2017).

Three hub genes, ADCY5, CXCL12, and NMU, were obtained by constructing PPI networks and using four different algorithms. ADCY5 is a member of the membrane-bound adenylate cyclase family, which converts adenosine triphosphate into cAMP and pyrophosphate (Vijiaratnam et al., 2019). Functional studies have shown that ADCY5 may affect glucose metabolism through glucose-coupled insulin secretion in human islets (Dupuis et al., 2010). Mutations in the ADCY5 gene can cause dyskinesia and dystonia (Vijiaratnam et al., 2019). CXCL12, a class of cytokines with chemotactic activity, plays an important role in physiological and pathological processes including hematopoiesis, angiogenesis, and inflammation (Bakogiannis et al., 2019; Döring et al., 2019; Mousavi, 2020). CXCL12 has been shown to activate and induce the migration of endothelial cells and most leukocytes, and exert biological effects under homeostatic and pathological conditions by interacting with its receptors, including atypical chemokine receptor 3 (ACKR3) and CXC chemokine receptor 4 (CXCR4), and binding to glycosaminoglycans (GAGs) in tissues and endothelium (Janssens, Struyf & Proost, 2018). NMU is a smooth muscle contractile polypeptide that is widely distributed in peripheral organs and the central nervous system (Martinez & O’Driscoll, 2015). Two specific endogenous receptors for NMU, NMU1R, and NMU2R, are widely distributed in animals and have different distribution patterns, demonstrating NMU’s diversity of physiological functions (Howard et al., 2000). NMU has been shown to have important physiological functions in the regulation of smooth muscle contraction, blood pressure, local blood flow, stress, and energy metabolism (Jarry et al., 2019). Additionally, we also predicted the TFs of differentially expressed lncRNAs. Studies have shown that the RHOXF1 is preferentially expressed in reproductive tissues (Borgmann et al., 2016). The SNAI2 is a downstream effector of the estrogen receptor alpha pathway and a key inducer of epithelial-to-mesenchymal transition (EMT). ZNF354C and TBX15 have been reported to be related to the occurrence of various cancers, including breast and ovarian cancer (Gozzi et al., 2016; Zang et al., 2017). The above-mentioned predicted differences in TFs may play a role in the pathogenesis of vaginal LD and are worthy of further exploration.

The limitations of this study need to be specifically discussed. Only differentially expressed lncRNAs in vaginal epithelial tissue were screened and validated. To gain a deeper understanding of lncRNA functions and roles in LD development, the expression of certain miRNAs targeted by lncRNAs should be further investigated, which may provide new information about the pathogenesis and diagnosis of LD. We plan to undertake these efforts in future functional studies.

## Conclusion

In this study, we report for the first time the profile of differentially expressed lncRNAs in LD. We found that a large number of lncRNAs are involved in LD pathogenesis. The GO and KEGG pathway and co-expression analysis results indicated that these differentially expressed lncRNAs may be related to the occurrence and development of LD. Further research is needed to explain the biological progress and molecular mechanism of dysregulated lncRNAs.

## Supplemental Information

10.7717/peerj.12485/supp-1 Supplemental Information 1 Fig. 2 Raw Data

## Additional Information and Declarations

### Data Availability

The following information was supplied regarding data availability: The data sets used and/or analyzed in this study are available at NCBI SRA: PRJNA646352.
